# Coping, Civilian Transition, and Gambling Harm Severity in UK Armed Forces Veterans

**DOI:** 10.1007/s10899-025-10451-6

**Published:** 2025-11-04

**Authors:** Adanma Ekenna, Dana Dekel, Hilary Engward, Lauren Godier-MacBard, Christopher Kay, Thomas Kersey, Matt Fossey, Simon Dymond

**Affiliations:** 1 Centre for Military Gambling Research, Swansea University, Singleton Campus, Swansea, SA2 8PP UK; 2 Veterans and Families Institute for Military Social Research, Anglia Ruskin University, Chelmsford, CM1 1SQ UK; 3 Department of Psychology, Reykjavík University, Menntavegur 1, Nauthólsvík, 101 , Reykjavík, Iceland

**Keywords:** Veterans, Problem gambling, Coping strategies, Civilian transition, Primary care

## Abstract

Veterans are prone to experiencing both mental health challenges and harm from gambling, yet little is known about the psychosocial factors that may increase risk. This study examined predictors of gambling harm severity in a sample of UK Armed Forces veterans with a focus on coping styles and reintegration into civilian employment. A cross-sectional survey was conducted among 414 former personnel using validated measures of gambling behavior, coping, and mental health symptoms including alcohol use. Of the 226 (54.6%) who had gambled in the past year, 3.1% had scores suggestive of problem gambling. Results showed that 46% of participants exhibited symptoms suggestive of posttraumatic stress disorder (PTSD), while 8.4% reported severe anxiety, 10.2% severe depression, and 11.1% high-risk alcohol use. Gambling harm severity was positively predicted by difficulties adapting to civilian employment and maladaptive coping strategies such as self-blame and substance use, while use of informational support was found to be protective. Given higher rates of primary care engagement among veterans, routine screening for gambling-related harm in such settings may improve early detection and intervention. The findings underscore the need for integrated mental health services that consider both psychological and social determinants of gambling harm severity in veterans during the military-to-civilian transition.

**Supplementary Information:**

The online version contains supplementary material available at 10.1007/s10899-025-10451-6.

## Introduction

Gambling and the harm it can cause are now widely regarded as a growing global public health challenge (Wardle et al., 2024). Gambling harm severity is often inferred along a continuum from low-risk, moderate-risk to problem gambling on the Problem Gambling Severity Index (PGSI) (Ferris & Wynne, 2001). At the extreme end of the harm continuum, problem or problematic gambling (PG) refers to gambling practices that create multiple problems that disrupt personal, family, financial, and employment circumstances (Wardle et al., 2024). Some populations may be at heightened vulnerability to experiencing harm from gambling, such as current and former members of the military. Indeed, among former military personnel (veterans), PG remains underreported and underrecognized compared to other psychiatric disorders (Metcalf et al., 2023).

Recent-year PG prevalence is estimated at 2.2–4.2%, with lifetime prevalence around 10% (Stefanovics et al., 2017; Westermeyer et al., 2005; Whiting et al., 2016). Some estimates suggest moderate-to-severe PG affects 31.97% of veterans - nearly double the civilian rate (van der Maas & Nower, 2021) - with veterans in one UK-based study by our group found to be up to ten times more likely to meet the criteria for PG than non-veterans (Dighton et al., 2018). These findings highlight an increased risk of gambling harm severity among veterans compared to the general population.

Veterans face unique risks due to trauma exposure and cultural transitions that increase vulnerability to risky behaviors, including PG (Fry et al., 2023). Military service is an all-encompassing lifestyle with strict cultural norms (McCaslin et al., 2021), specialized roles with limited flexibility (Redmond et al., 2015), combat exposure, high-stress environments, and operational risks that distinguish them from civilian jobs (Adler & Castro, 2013). Therefore, the transition to civilian life leads to increased engagement in high-risk behaviors, including gambling (Ashley & Milam, 2017; Thandi et al., 2015).

These challenges are framed by the military-to-civilian transition (MCT) which conceptualizes the transition as a multidimensional process involving psychological, social, and occupational adaptation (Pedlar et al., 2019). It requires adaptation to an unfamiliar culture, employment, healthcare, relationships, and housing challenges (Pedlar et al., 2019). MCT highlights that successful transition requires managing identity disruption, reconciling military and civilian norms, and developing adaptive coping strategies in new social and occupational contexts (Karre et al., 2024; Kintzle & Castro, 2018; Pedlar et al., 2019). Difficulties in this adjustment—such as friction in adopting civilian roles (Walker, 2013), loss of structured support, and changes in peer networks—can increase susceptibility to mental health problems and engagement in high-risk behaviors (Sharp et al., 2015). Psychiatric issues often double within the first five years post-transition (Van Hooff et al., 2018a), underscoring the critical need to understand how coping and adaptation processes influence post-service outcomes, including gambling harm.

The complex relationship between military experiences and post-service adjustment challenges increases veterans’ susceptibility to gambling, compounded by common mental health disorders (CMD) (Dighton et al., 2018; Grubbs et al., 2018; Hitch et al., 2023; Metcalf et al., 2023; Moore & Grubbs, 2021; Rhead et al., 2022). Alcohol use, common among veterans (Jalilian-Khave et al., 2024), is more severe among those dealing with gambling disorder (Grubbs et al., 2024; Stefanovics et al., 2023b) and heightens the impact of trauma and CMD (Stefanovics et al., 2024). Social factors, such as strong family support and chronic homelessness (Stefanovics et al., 2023a; Weisenhorn et al., 2016), play a role in gambling harm severity, with loneliness emerging as an underexplored risk factor. Indeed, loneliness, a major public health concern, is linked to poor mental health (Biddle et al., 2005; Heron et al., 2022; Leigh-Hunt et al., 2017), yet is rarely assessed among UK veterans. High levels of loneliness have been reported among UK veterans (The Royal British Legion, 2018; Williamson et al., 2023a), linked to higher alcohol misuse (Williamson et al., 2023b), and mental and physical health issues (Straus et al., 2022).

Given these challenges, coping effectively with stress is crucial for both service personnel and veterans. Veterans exhibit a greater tendency to utilize maladaptive coping strategies when managing stress (Fegley, 2024; Korem et al., 2023). Evidence shows that veterans’ resilience is positively predicted by a longer time-in-service, increased use of humor, and a lower reliance on self-blame as coping strategies (Rice & Liu, 2016). Like veterans, individuals who experience PG are more likely to employ maladaptive coping strategies compared to those who do not gamble (Getty et al., 2000). Additionally, avoidant coping strategies have been found to have a stronger positive correlation with the severity of problem gambling than non-avoidant coping strategies (Yi & Kanetkar, 2011).

Building on our previous work (Dighton et al., 2022; Harris et al., 2023), the present exploratory study sought to investigate the role of aforementioned psychosocial vulnerabilities, particularly loneliness and coping strategies, on gambling among veterans. We conducted a study of UK veterans examining their sociodemographic characteristics, recent gambling behaviors, perceived loneliness and coping strategies as predictors of gambling harm severity. We also investigated potential interactions between gambling harm and healthcare utilization and post-service employment opportunities.

## Method

### Participants and Procedure

A cross-sectional online survey of Armed Forces veterans was conducted between January and October 2024. To participate, respondents had to be former members of HM Armed Forces, UK residents, and provide an accurate Armed Forces service number. Recruitment was conducted via social media (e.g., Facebook, LinkedIn, X) and emails and flyers circulated among national veterans’ charities and support organizations. Following pilot testing, the survey was hosted on Qualtrics and distributed online.

The survey recorded 1,450 engagements; quality control measures subsequently filtered out incomplete or questionable responses, such as failure to complete the socio-demographic and military demographics questions (*n* = 998), failure to complete all scales (*n* = 27), and incomplete responses erroneously recorded as complete (*n* = 11). The final sample consisted of responses from *n* = 414 veterans (mean age = 65.3 years; SD = 9.5; 98.8% male) of whom 226 reported a recent gambling history. A post-hoc sensitivity analysis (Faul et al., 2007) with *n* = 226, ⍺ =0.05 and power (1 – β) = 0.8 indicated that a sample size of 92 could detect a Cohen’s f = 0.15 and a critical F = 2.32 (numerator DF = 5, denominator DF = 86) in predicting gambling harm severity from five mental health variables. Ethical approval was obtained from the School of Psychology Research Ethics Committee, Swansea University (Approval number: 1 2024 8399 8380).

## Measures

The study assessed sociodemographic characteristics, military demographics, gambling participation and severity, mental health (anxiety, depression, posttraumatic stress disorder (PTSD), loneliness, coping styles, alcohol use), employment, benefits and debt, and healthcare service utilization among UK veterans.

### Sociodemographic Characteristics

Respondents reported their date of birth, sex, gender, ethnicity, relationship status, highest qualification, accommodation type, and living situation (e.g., alone, with family).

### Military Demographics

Veterans provided their service number, enlistment and discharge dates, years served, branch, trade, discharge type, rank at discharge, number of deployments, and 37 deployment locations (see Supplementary Table 1).

### Gambling Engagement

Veterans were asked whether they had gambled in the past year and, if they had, to identify one or more of 23 gambling activities (see Supplementary Table 2).

### Gambling Harm Severity

Gambling harm severity of participants who reported past-year gambling was assessed using the 9 item Problem Gambling Severity Index (PGSI; Ferris & Wynne, 2001). Respondents rated items such as, “Have you bet more than you could really afford to lose?” on a 4-point scale from ‘Never’ (0) to ‘Almost Always’ (3). Total scores classified gambling behavior as: 0 (non-problem gambling), 1–2 (low-risk gambling), 3–7 (moderate-risk gambling), and 8+ (problem gambling). Internal consistency was excellent (α = 0.900).

## Mental Health Variables

*Anxiety.* Anxiety was assessed using the generalized anxiety disorder seven-item scale (GAD-7; Spitzer et al., 2006). Respondents rated how often they experienced anxiety symptoms on a scale from 0 (‘not at all’) to 3 (‘almost every day’). Scores ranged from 0 (normal anxiety) to 21 (severe anxiety). Internal consistency was excellent (α = 0.946).

*Depression.* Depression severity was assessed using the patient health questionnaire nine-item scale (PHQ-9; Kroenke et al., 2001). Respondents rated depressive symptoms over the past two weeks, with scores 0–4 for no depression or mild depression to ≥ 20 for severe depression. Internal consistency was excellent (α = 0.939).

*PTSD.* The PTSD Checklist for DSM-5(Diagnostic and Statistical Manual of Mental Disorders, Fifth Edition) (PCL-5; Blevins et al., 2015) is a 20-item self-report measure assessing symptoms of PTSD based on DSM-5 criteria. Respondents rated each of the 20 items on a scale from 0 (‘not at all’) to 4 (‘extremely’), with scores ranging from 0 to 80. Scores ≥ 33 indicated clinically significant PTSD (Bovin et al., 2016; Kruger-Gottschalk et al., 2017; Rosendahl et al., 2019). The reliability was excellent (α = 0.970). The Life Events Checklist for DSM-5 (LEC-5; Weathers et al., 2013), a 17-item measure, assessed trauma exposure and was used in conjunction with the PCL-5. Higher scores indicate greater trauma exposure.

*Loneliness.* Loneliness was assessed using the three-item University of California, Los Angeles (UCLA) Loneliness Scale (Version 3; Hughes et al., 2004), which includes three items rated from 1 (‘hardly ever’) to 3 (‘often’). Higher scores indicated greater loneliness. Internal consistency was excellent (α = 0.923).

*Coping styles.* The Brief COPE (Carver, 1997) is a 28-item measure used to evaluate how individuals respond to stress, using 14 subscales which can be grouped into problem-focused, emotion-focused and avoidant coping (Carver et al., 1989). Respondents rated how often they used each strategy on a 4-point scale (1 = ‘not at all’, 4 = ‘a lot’). The scoring was by summing two items per subscale with a range of 2–8 (Carver, 1997) or by taking the mean score of each of the 14 subscales (Cooper et al., 2006). Higher mean scores indicate greater use of that coping strategy. Reliability was excellent (α = 0.938).

## Other Measures

*Alcohol use*. The Alcohol Use Disorders Identification Test-Concise (AUDIT-C; Bradley et al., 2003) screened for hazardous drinking. Respondents answered three questions on frequency, quantity, and binge drinking. Higher scores indicated greater risk. The reliability was good (α = 0.799).

*Employment*,* benefits and debt*. Respondents were asked about their employment status, state benefits, monthly income, ease of finding employment after service, duration of job search, experience in adapting from military training to civilian job training, recognizable military qualifications by employers, transferable military skills in present job, debt type, debt quantification and cancellation (Harris et al., 2023).

*Healthcare services utilization*. The Client Service Receipt Inventory (CSRI; Beecham & Knapp, 2001) measured National Healthcare Service (NHS) and social service utilization including primary and secondary healthcare services.

### Data Analysis

Descriptive statistics were reported as percentages, means and standard deviations. Normality of continuous variables was assessed using skewness, kurtosis, and the Shapiro-Wilk test. Several variables including all mental health factors, PGSI, age and service years, showed significant deviations from normality (*p* <.001), leading to the use of log transformations to improve distributional properties. Categorical variables were analyzed using frequency distributions and chi-square tests, where appropriate. Pearson correlation analyses were conducted to examine associations between the mental health factors and PGSI scores. Multiple linear regressions were performed to identify socio-demographic, military demographic and mental health predictors of gambling harm severity. Both Bayesian and frequentist regressions were employed, as the approaches are complementary in addressing model uncertainty and improves small-sample performance (Celeux et al., 2012; Wakefield, 2013). Multicollinearity was assessed using variance inflation factors and tolerance values, which remained below 5 and above 0.1 respectively, indicating no violation of assumptions. To enhance the robustness of results and account for potential assumption violations, bootstrapped confidence intervals were computed. Reference groups for categorical predictors were selected based on theoretical and practical considerations, with models incorporating living situations, benefits, military demographics, service branch, adaptation to civilian jobs, and coping styles.

Bayesian analyses were conducted using JASP’s default JZS priors (*r* =.354), and sensitivity analyses with alternative priors produced consistent posterior estimates and Bayes Factors. Bayes Factors (BF_10_) were used to assess the strength of evidence for the alternative hypothesis relative to the null hypothesis, where values greater than 1, less than 1, and equal to 1 represent greater evidence for the alternative hypothesis, greater evidence for the null hypothesis, and no evidence for either hypothesis, respectively (Lee & Wagenmakers, 2013). All statistical tests were performed at α = 0.05. All statistical analyses were conducted using JASP Version 0.19.3 (JASP Team, 2024) and IBM SPSS V.29 (IBM Corporation, 2024).

## Results

### Sociodemographic Characteristics

Table 1 shows the sociodemographic profile of the sample. Most respondents were male (98.8%) with a mean age of 65.3 years (SD = 9.5, Range = 27–84), resided in England, and were of white ethnicity. Veterans commonly held at least one GCSE qualification, were in their first or later marriage, lived with a spouse or partner and owned their homes.

**Table 1:** Socio-demographic characteristics of the sample

Variables	*N*	Percent
**Age (years)**		
< 60	100	24.1
60–74	226	54.6
75+	88	21.3
**Sex**		
Male	409	98.8
Female	5	1.2
**Gender**		
Male	408	98.6
Female	6	1.4
**Residence**		
England	335	80.9
Northern Ireland	10	2.4
Scotland	41	9.9
Wales	28	6.8
**Ethnicity**		
White	409	98.8
Mixed or Multiple ethnic groups	2	0.5
Asian or Asian British	0	0.0
Black, Black British, Caribbean or African	1	0.2
Other	2	0.5
**Highest educational qualification**		
No formal qualification	62	15.0
Entry certificate	17	4.1
GCSE D-G	48	11.6
GCSE A*-C	65	15.7
AS/A level	30	7.2
Certificate of HE	59	14.3
Bachelor’s degree	81	19.6
Master’s degree	48	11.6
Doctorate	4	1.0
**Relationship**		
Single	32	7.7
In a relationship	26	6.3
Co-habiting	12	2.9
Married-first and only marriage	106	25.6
Married-second or later marriage	9	2.2
Separated	37	8.9
Divorced	31	7.5
Widowed	3	0.7
Other	158	38.2
Other relationship	*n* = 158	
Divorced now co-habiting new partner, Poly/ENM, Married 3rd marriage	3	1.8
Unspecified	155	98.2
Home arrangement*		
Live alone	96	19.8
Children under 18	38	7.9
Children over 18	43	8.9
Spouse/partner	290	59.9
Other family	11	2.3
Parent(s)	1	0.2
Non-family	5	1.0
Other home arrangements	*n* = 5	
Daughter’s boyfriend	1	20.0
Wife	2	40.0
Homeless	1	20.0
Sister	1	20.0
**Accommodation**		
Owner occupied flat or house	269	65
Rented from local authority	61	14.7
Privately rented flat or house	39	9.4
Domestic/family	32	7.7
Other	7	1.7
Sheltered housing	2	0.5
Independent living	2	0.5
Supported lodging	1	0.2
Homeless	1	0.2

*Multiple options selected

## Military Demographics

Table 2 shows the military demographic characteristics of the sample. Most respondents had served between 1 and 10 years, were discharged 26 or more years ago, left at the end of their engagement, served in the Army, and held non-commissioned officer rank at discharge. They typically deployed between 1 and 10 times with an average tour length of 4 to 6 months. The commonest deployment locations were Falkland Islands, Belize and the 2nd Gulf War (Supplementary Table 1).

**Table 2:** Military demographics

Variables	*N*	Percent
**Enlistment date**		
1950–1969	113	27.3
1970–1989	247	59.7
> 1989	48	11.6
**Service years**		
1–10	154	37.2
11–20	102	24.6
21–30	114	27.5
> 30	44	10.6
Service branch*		
Army/Army Reserves	304	66
Royal Navy/Marines	84	18
RAF/RAF reserves	75	16
**Rank at discharge**		
NCOs/other ranks	370	89.4
Commissioned officers	44	10.6
**Discharge date**		
1960–1979	75	18.4
1980–1999	215	52.7
2000–2019	98	24.0
2020–2024	20	4.9
**Type of discharge**		
Medical	27	6.5
At own request (PVR)	132	31.9
End of engagement	198	47.8
Administrative/Compulsory/Redundancy	35	8.4
Other	22	5.3
**Deployment frequency**		
0	26	6.4
1–10	312	77.2
11–20	57	14.1
20+	9	2.2
**Average operational tour length**		
Less than 1–3 weeks	19	4.6
1–3 months	46	11.2
4–6 months	172	41.8
7–12 months	64	15.6
Other	110	26.8
**Deployment locations**		
Falkland Islands	101	34.6
Belize	68	23.3
2nd Gulf (2003–2011)	61	20.9
Bosnia	60	20.5
Hong Kong	55	18.8
1 st Gulf (1991)	54	18.5
Singapore	41	14.0
Afghanistan 2001to 2021	38	13.0
Kenya	37	12.7
Kosovo	36	12.3
Aden	29	9.9
Germany	16	3.9

*Multiple options selected

### Gambling Engagement and Severity

Of 414 veterans, 226 (54.6%) reported recent gambling and were included in the analyses of gambling harm severity and mental health variables. Table 3 shows that National Lottery tickets (89.3%), National Lottery online (51.6%) and tickets for other charity lotteries (40.0%) were the most common activities reported out of 24 (Supplementary Table 2).

**Table 3:** Gambling activities and severity

Variables	*N*	Percent
Gambling activity*		
Tickets for National Lott*ery*	201	89.3
Tickets for National Lottery on*line*	116	51.6
Tickets for other charity lotte*ries*	90	40.0
Tickets for other charity lotteries online	42	18.7
National Lottery scratch cards	42	18.7
Tickets for National Lottery in person	41	18.2
Betting on sports and/or racing online or in app	36	16.0
National Lottery online instant win games	21	9.3
Fruit and/or slots in person	16	7.1
Betting on sports and/or racing in person	15	6.7
Bingo played at avenue	9	4.0
Tickets for other charity lotteries in person	7	3.1
Other scratch cards	7	3.1
Other online instant win games	7	3.1
Bingo played online or in app	6	2.7
Betting on outcome events online	5	2.2
Private betting such as with friends	5	2.2
Fruit and/or slots online or in app	4	1.8
Football pools	1	0.4
**Gambling severity**		
Non-problem gambling (0)	168	74.3
Low-risk gambling (1–2)	27	11.9
Moderate-risk gambling (3–7)	24	10.6
Problem gambling (8–27)	7	3.1

*Multiple responses

The mean PGSI score was 0.98 (SD 2.57). Out of 226 respondents, most experienced non-problem gambling 168 (74.3%), while 27 (11.9%) had scores indicative of low-risk, 24 (10.6%) moderate-risk, and 7 (3.1%) had scores suggestive of problem gambling (Table 3).

### Mental Health

Table 4 presents the descriptive statistics of all mental health variables. Most respondents reported minimal anxiety 131 (58.0%), followed by mild anxiety 56 (24.8%), moderate anxiety 20 (8.8%), and severe anxiety 19 (8.4%). Almost half (43.8%) of the respondents reported no depression, 60 (26.5%) mild 24 (10.6%), moderate 20 (8.8), moderately severe 20 (8.8%), and 23 (10.2%) with severe depression. The final PHQ-9 item is “thoughts that you would be better off dead or of hurting yourself in some way.” Within our sample, 13 (6%) of respondents reported having those thoughts nearly every day, 16 (7%) more than half the day, 27 (12%) over several days, and 170 (75%) not at all.

**Table 4:** Descriptive statistics and pearson’s correlation of mental health factors and gambling harm severity

	Mean (SD)	95% C.I.	1	2	3	4	5	6
1. PGSI	0.98 (2.57)	0.65–1.32	—					
2. Anxiety	4.92 (5.65)	4.18–5.66	0.29**	—				
3. Depression	7.42 (7.34)	6.46–8.38	0.30**	0.85**	—			
4. PTSD	17.13 (19.20)	14.62–19.65	0.25**	0.73**	0.71**	—		
5. Loneliness	5.58 (2.21)	5.29–5.87	0.30**	0.70**	0.75**	0.57**	—	
6. Alcohol use	1.20 (3.27)	1.55–2.41	0.15*	0.10	0.12	0.12	0.10	—

* *p* <.05, **p <.01. All variables were log-transformed prior to analysis. Categorical counts for each variable: Anxiety: Minimal 131 (58.0%), Mild 56 (24.8%), Moderate 20 (8.8%), Severe 19 (8.4%). Depression: None 99 (43.8%), Mild 60 (26.5%), Moderate 24 (10.6%), Moderately severe 20 (8.8%), Severe 23 (10.2%). PTSD: Below threshold 180 (79.6%), Above threshold 46 (20.4%). Loneliness: Low 112 (49.6%), Moderate 56 (24.8%), High 58 (25.7%). Alcohol use: Low 150 (66.4%), Moderate 51 (22.6%), High 0 (0.0%), Very high 25 (11.1%).

Almost half (46%) of the respondents reported symptoms suggestive of PTSD, with an average of 6.1 traumatic events exFsupplperienced or witnessed. Unwanted sexual experience was the most common form of traumatic event experienced (Supplementary Table 3). Most of the respondents had experienced or witnessed 0 to 10 traumatic events and those with high trauma exposure (11–21 events) were more likely to have symptoms suggestive of PTSD (OR = 1.65, 95% CI [0.93, 2.37], Cramer’s V = 0.318).

Loneliness was scored across three categories: low-level (112; 49.6%), moderate level (56; 24.8%), and high-level loneliness (58; 25.7%). Overall, half of the respondents experienced moderate to high levels of loneliness.

Table 4 also presents the Pearson correlation analyses results. PGSI scores were moderately positively associated with anxiety (*r* =.29, *p* <.001), depression (*r* =.30, *p* <.001), PTSD symptoms (*r* =.25, *p* <.001), and loneliness (*r* =.30, *p* <.001), and showed a weaker positive association with alcohol use (*r* =.15, *p* =.025).

When coping scores were summed, the highest mean score was for emotion-focused coping, followed by problem-focused coping and avoidant coping (Table 5). Disaggregated mean scores within the three-order subscales show that the highest mean scores were for humor and self-blame in emotion-focused coping, use of informational support in problem-focused coping, and self-distraction in the avoidant coping category. Of all the subscales, self-blame had the highest frequency of respondents with 23 (10.2%) reporting “I have been doing this a lot”.

**Table 5:** Multiple linear regression of sum scores of problem-focused, emotion-focused and avoidant coping styles and gambling severity

	Mean (SD) Average score 1–4	B	SE	β	T	*P*
Problem-focused coping (Total score 8–32)	14.5 (6.3)					
Use of informational support	2.5 (0.5)	− 0.25	0.10	− 0.17	−2.48	0.014
Active coping	2.0 (0.5)	0.04	0.14	0.02	0.52	0.606
Planning	1.5 (0.5)	0.06	0.17	0.16	0.61	0.545
Positive reframing	1.0 (0.0)	− 0.06	0.16	− 0.03	−0.71	0.477
Emotion-focused coping (Total score 10–35)	20.6 (6.99)					
Humor	3.5 (0.5)	− 0.03	0.15	− 0.03	−0.39	0.694
Self-blame	3.5 (0.5)	0.47	0.10	0.36	4.63	< 0.001
Emotional support	2.25 (0.75)	− 0.02	0.15	0.003	−0.23	0.821
Venting	1.0 (0.0)	− 0.17	0.17	− 0.23	−1.81	0.059
Acceptance	0.75 (0.75)	0.02	0.14	0.02	0.21	0.838
Religion	0.5 (0.5)	− 0.01	0.16	− 0.01	−0.20	0.839
Avoidant coping (Total score 9–38)	12.7 (5.1)					
Self-distraction	2.75 (1.25)	0.04	0.13	0.06	0.52	0.604
Substance use	2.5 (0.0)	0.23	0.10	0.17	2.39	0.018
Denial	2.25 (0.5)	0.10	0.15	0.12	1.16	0.247
Behavioral disengagement	2.25 (0.25)	0.05	0.16	0.06	0.55	0.586
Model summary R R^2^ Adjusted R^2^ F (1,224) *p*	0.41 0.17 0.156 14.76 < 0.001					

Note. *B = unstandardised; SE* = Standard Error; *β* = standardised; *T* = t value; *P* = p value

Alcohol use scores indicated that 150 (66.4%) were deemed low risk, 51 (22.6%) moderate risk, and 25 (11.1%) deemed to be at very high risk.

### Employment, Benefits and Debt

Most respondents were employed full-time, not in receipt of state benefits, earned between £1,500 to £1,900 monthly, secured employment within three months after leaving the Armed Forces, and found adaptation to civilian job training moderately difficult (Supplementary Table 4). Most reported the use of their military skills in their current roles while some (29.9%) reported that their military qualifications were occasionally recognized by their employers. Regarding debt, only 0.9% had no debt, with overdrafts being the most reported. Most debt amounts ranged from £1,000 to £5,000 (Supplementary Table 5).

### Healthcare Services Utilization

Most respondents used general practice (GP) services with 107 (28%) using in-person consultations, and over half of that had 1–5 GP contacts in the previous three months, with an average of 2.87 contacts (*SD* = 3.99) (Supplementary Table 5). Most (81%, *n* = 191) had no inpatient healthcare service use, with a mean of 1.1 days (*SD* = 0.3) among those who did. Community nurse services, with an average of 1.4 contacts (*SD* = 0.7), and self-help groups were the commonly accessed non-hospital-based care and additional services, respectively. The respondents rated their overall health perception at 6.31 (*SD* = 2.17) (Supplementary Table 6).

### Predictors of Gambling Harm Severity

*Sociodemographic predictors* Multiple linear regression revealed that older age predicted lower gambling harm severity (ΔR² = 0.05, F (1,224) = 13.75, *p* <.001, BF₁₀ = 1.000), as did longer time since leaving service (ΔR² = 0.02, F (3,203) = 2.49, *p* =.016, BF₁₀ = 0.46). Positive predictors included living with children under 18 (ΔR² = 0.07, R²_adj = 0.04, F(7,218) = 2.371, *p* =.001, BF₁₀ = 1.000), receiving state benefits (ΔR² = 0.04, F(5,220) = 2.22, *p* =.038), not receiving a state pension (ΔR² = 0.04, F(5,220) = 2.22, *p* =.003), and severe difficulty adapting to civilian job training (ΔR² = 0.055, F(2,223) = 7.55, *p* <.001, BF₁₀ = 1.227).

*Mental health predictors*. The multiple linear regression model of mental health factors was statistically significant, *F* (0,5) = 5.87, *p* <.001, accounting for 12% of the variance in gambling harm severity (R^2^ = 0.12, Cohen’s *f*^2^ = 0.136). None of the variables significantly predicted gambling harm severity (Supplementary Table 7).

The model examining coping styles (sum scores of 14 Brief COPE subscales) predictive of gambling harm severity was significant, *F* (3,220) = 14.76, *p* <.001 (Table 5). Self-blame (β = 0.38, *p* =.015, BF₁₀ = 1.000) and substance use (β = 0.22, *p* =.018, BF₁₀ = 0.012) emerged as significant positive predictors, while use of informational support (β = −0.25, *p* =.014, BF₁₀ =1.1 × 10^− 4^) was a significant negative predictor of gambling harm severity, respectively. In another model (R^2^_adj_ = 0.13, F (3,220) = 10.8, *p* <.001) of the three higher-order subscales, only the avoidant coping strategies were found to be statistically significant (β = 0.70, *p* <.001, BF₁₀ = 1.000).

## Discussion

This study presents findings on gambling harm severity and mental health among a sample of UK Armed Forces veterans. Framed through MCT, our findings indicate that veterans who have trouble adapting to civilian employment and rely on maladaptive coping strategies, such as self-blame or avoidant styles, are more likely to experience higher gambling harm severity. This aligns with MCT, which suggests that inadequate adaptation to civilian roles and stressors can exacerbate vulnerability to risky behaviors. Moreover, the protective effect of informational support observed in this study supports MCT’s proposition that social and informational resources facilitate smoother transitions and buffer against maladaptive behaviors. Veterans’ engagement with primary care services further illustrates opportunities to support identity and role adjustment during the transition period.

The prevalence of problem gambling identified in our sample (3.1%) aligns closely with international findings, reinforcing the notion that veterans are at heightened risk across diverse settings. Similar prevalence rates have been reported, different nomenclatures in reporting considered, including 4.6% PG and 8.8% for at-risk problem gambling (ARPG) in Australia (Metcalf et al., 2022), 4.2% for ARPG (Whiting et al., 2016), and 2.2% ARPG in another US veteran sample (Stefanovics et al., 2017). Lifetime prevalence among UK veterans has also been recorded at 3.6% (Dighton et al., 2018). These rates are notably higher than those observed in the general UK population, where PG is stable at 0.3% (Gambling Commission, 2023). The consistency of these elevated rates across international contexts (Biddle et al., 2005; Westermeyer et al., 2013) suggests that PG is a universal concern within veteran populations.

The present work showed that, consistent with UK studies (The Royal British Legion, 2018), 25.7% of veterans experienced a high level of perceived loneliness, which was higher than comparable U.S. samples (Kuwert et al., 2014; Na et al., 2022; Straus et al., 2022). Such global differences may reflect cultural factors, service-related experiences, or the structure of post-service support systems (e.g., Veterans’ Affairs mental health service vs. NHS’s veteran mental health and wellbeing service, Op COURAGE) (National Health Service, 2020). Collectively, our results on common mental health disorders (CMD) are consistent with existing literature indicating a high prevalence of CMD among military populations (Goodwin et al., 2015), though reported rates vary across studies (Campbell et al., 2024; Finnegan & Randles, 2023; Rhead et al., 2022; Stevelink et al., 2018), likely due to methodological and population differences. Alcohol misuse rates mirrored other UK studies (Finnegan & Randles, 2023; Rhead et al., 2022; Stevelink et al., 2018), but were lower than US figures (Davis et al., 2017; Fuehrlein et al., 2016). Correlations seen in bivariate analyses may have lost significance in multivariate models due to shared variance among predictors.

Veterans relied on humor, self-blame, and self-distraction as coping styles. Humor has been found to help military personnel manage unique challenges (Maxwell, 2003; Yoshimura et al., 2024). However, self-blame and self-distraction—both maladaptive—are linked to CMD and psychological distress (Mazzulo, 2018; Romero et al., 2020). Therefore, our findings underscore the complex role of coping mechanisms suggesting maladaptive strategies are linked to greater psychological distress. Self-blame and substance use positively predicted gambling harm severity, while use of informational support was found to be protective. The link between substance use and gambling among veterans is well-documented (Davis et al., 2017; Grubbs & Chapman, 2019; Grubbs et al., 2024; Stefanovics et al., 2017; Stefanovics et al., 2024), however, associations involving self-blame and informational support are less explored. Informational support has been identified as a protective factor against CMD among serving personnel (Akinlose et al., 2024). Additionally, building on prior evidence of a positive correlation between avoidant coping and problem gambling (Yi & Kanetkar, 2011), our study demonstrates that avoidant coping positively predicts gambling harm severity. Evidence indicates that veterans are often motivated to gamble as a means to avoid or escape psychological distress (Dighton et al., 2022), this broadens the understanding that avoidant-based coping is both a response to underlying vulnerability and a contributor to the onset and maintenance of gambling harm severity within this population.

Difficulty adapting to civilian employment was a significant predictor of gambling harm severity in veterans. Psychological stress associated with shedding a military identity and adopting a new one in civilian settings (Hunter-Johnson et al., 2020; Minnis, 2020; Van Hooff et al., 2018b; Walker, 2013) with challenges from a “culture clash” with colleagues in civilian jobs, ill-feelings for having to start at entry roles or feelings of discrimination by employers against veterans (Keeling et al., 2018; Oh et al., 2021) have been reported. These challenges are central to MCT, which frames the transition process as a multidimensional adjustment encompassing occupational, social, and personal identity domains (Pedlar et al., 2019). Our finding reinforces evidence that adaptation difficulties are a meaningful stressor during the military-to-civilian transition (Harrod et al., 2017; Krigbaum et al., 2020; Shue et al., 2021).

Veterans in our study used primary care more frequently than the general population (National Health Service Digital, 2007). This aligns with data showing higher primary care use among older adults, (Mukhtar et al., 2018). Near universal (98%) GP registration among UK and Australian veterans is documented (Office for National Statistics, 2024; Roughead et al., 2010). Increased utilisation likely reflects veterans’ older age, co-morbidities, and a responsive healthcare system (Finnegan et al., 2022). Given this high engagement, primary care offers a strategic opportunity for implementing gambling harm screening (Roberts et al., 2019; Roberts et al., 2025), particularly as veterans are identified as an at-risk group (National Institute for Health and Care Excellence, 2025).

The positive predictive relationships between self-blame, substance use, and gambling harm severity highlight the need to address psychological distress through adaptive coping strategies. Targeting emotional regulation and trauma-related symptoms could reduce gambling vulnerability. Transition difficulties, consistent with MCT, further emphasize the need for tailored veteran support during this high-risk period. Trauma-informed care that integrates mental health and gambling support is essential. Gambling screening within services like UK’s Op COURAGE service (National Health Service, 2020) aligns with national strategies (National Health Service, 2019; National Institute for Health and Care Excellence, 2025; Office for Veterans’ Affairs, 2022). Similar international programs exist (Department of Veterans’ Affairs, 2024; Veterans Affairs, 2025). A multi-agency response is vital to reduce gambling harm and promote veteran well-being.

This study offers a unique perspective by focusing on an older veteran sample and addressing critical social issues such as perceived loneliness and transition to civilian life. Our findings align with NICE guidelines (National Institute for Health and Care Excellence, 2025), reinforcing the classification of veterans as an at-risk group and highlighting the need for screening measures that incorporate loneliness assessments. This study is subject to some limitations which need to be viewed within context. Common to all cross-sectional designs, any causal interpretations are precluded, and the self-selected nature of the sample introduces the possibility of selection bias. Although post hoc sensitivity analysis suggests adequate statistical power, the sample was predominantly male and comprised mainly of British Army veterans, which may restrict the generalizability of the findings across broader veteran populations. Additionally, the study did not assess gambling motivations, which may have provided further context for understanding the mechanisms underlying gambling harm severity.

### Implications

This study highlights the need for veteran-specific interventions addressing gambling harm, mental health, and civilian transition challenges. Difficulties in adapting to civilian employment and reliance on maladaptive coping strategies underscore that gambling behavior is often intertwined with broader adjustment stressors during MCT. Recent guidelines advocate targeted screening and early intervention for high-risk groups, including veterans (Department for Culture Media & Sport, 2023; National Institute for Health and Care Excellence, 2025). The development of targeted prevention and intervention programs need to be prioritized and addressed taking into consideration the unique social and psychological stressors faced by this population. Primary care settings should incorporate routine screening for gambling behaviors within the veteran populations, particularly in those exhibiting signs of social isolation and challenges in adapting to civilian life. By recognizing gambling not as an isolated behavior but as a symptom of broader adjustment difficulties, practitioners and policymakers can more effectively allocate resources to support long-term recovery.

## Conclusions

Difficulties associated with the MCT, including employment adaptation challenges and reliance on avoidant-based coping strategies were key psychosocial factors contributing to gambling harm severity among this sample of UK veterans. Use of informational support was found to be protective, highlighting the importance of targeted resources during the transition period. These findings emphasize the need for routine screening for gambling behaviors in primary care settings, where veterans are more likely to present, should be undertaken and help support early intervention. Overall, these results underscore the need for integrative, veteran-specific mental health services that account for both clinical and social determinants of health within the broader context of MCT.

## Supplementary Information

Below is the link to the electronic supplementary material.

Supplementary Material 1

## Data Availability

The data described in this article are openly available in the Open Science Framework at https://osf.io/4twx2/?view_only=5e12707fc7974e9291683781220b030c.
