# Supplementary material for: Coping, Civilian Transition, and Gambling Harm Severity in UK Armed Forces Veterans
Source: J Gambl Stud. 2025 Nov 4;42(1):199–218. doi: 10.1007/s10899-025-10451-6 (PMC13009049; doi:10.1007/s10899-025-10451-6)
Supplement: Supplementary file 1 — Supplementary Material 1 [file 10899_2025_10451_MOESM1_ESM.docx]

## Supplementary Tables

[**Supplementary Table 1** List of deployment locations 1](#_Toc201649470)

[**Supplementary Table 2** List of gambling activities 2](#_Toc201649471)

[**Supplementary Table 3** Life events checklist that happened to or witnessed by the veterans 3](#_Toc201649472)

[**Supplementary Table 4** Employment and benefits 4](#_Toc201649473)

[**Supplementary Table 5** Debts 7](#_Toc201649474)

[**Supplementary Table 6** Healthcare utilisation 9](#_Toc201649475)

**Supplementary Table 7** Multiple linear regression of mental health factors and gambling harm severity…..…………………………………………………………………………………...…..11

**Deployment locations reported by veterans**: This table lists global locations where veterans reported deployment, including conflict and non-conflict zones.

**Supplementary Table 1** List of deployment locations

| Northern Ireland | Dafoe |
| --- | --- |
| Falkland Islands | Afghanistan (2001-2021) |
| Bosnia | Aden |
| Kosovo | Columbia |
| 1^st^ Gulf (1991) | Chile |
| 2^nd^ Gulf (2003-2011) | Somalia |
| Rwanda | South Africa |
| Kenya | Rhodesia |
| Cyprus (Non-R&R) | Egypt |
| Belize | Libya |
| Sierra Leone | Russia |
| Lebanon | Grenada |
| World War II | Ivory Coast |
| Cuban crisis | Guyana |
| Macedonia | Oman |
| Pakistan | Yemen |
| Iraq/Iran conflict (1997) | Vietnam |
| Hong Kong | East Timor |
| Singapore | Other |

**Types of gambling activities engaged in by veterans**: An overview of gambling formats accessed in the past 12 months, ranging from lottery tickets to casino games and online betting.

**Supplementary Table 2** List of gambling activities

| Tickets for National Lottery | Betting on outcome events online |
| --- | --- |
| Tickets for National Lottery online | Betting on outcome events in person |
| Tickets for National Lottery in person | Bingo played online or in app |
| Tickets for other charity lotteries | Bingo played at a venue |
| Tickets for other charity lotteries online | Casino games online or in app |
| Tickets for other charity lotteries in person | Casino games played in a casino |
| National Lottery scratchcards | Casino games played on a machine in a venue |
| Other scratchcards | Fruit and/or slots online or in app |
| National Lottery online instant win games | Fruit and/or slots in person |
| Other online instant win games | Football pools |
| Betting on sports and/or racing online or in app | Private betting, such as, with friends |
| Betting on sports and/or racing in person | Another form of gambling in the last 12 months |

**Supplementary Table 3** Life events checklist that happened to or witnessed by the veterans

| Variable | N | Percent |
| --- | --- | --- |
| Life events that happened to the respondents |  |  |
| Natural disaster | 21 | 2.7 |
| Fire or explosion | 56 | 7.2 |
| Transportation accident | 109 | 14.1 |
| Serious accidents at work, home, or during | 55 | 7.1 |
| Exposure to toxic substance | 40 | 5.2 |
| Physical assault | 103 | 13.3 |
| Assault with a weapon | 24 | 3.1 |
| Sexual assault | 29 | 3.8 |
| Other unwanted or uncomfortable sexual experience | 116 | 15.0 |
| Combat or exposure to a warzone | 9 | 1.2 |
| Captivity | 82 | 10.6 |
| Life-threatening illness or injury | 11 | 1.4 |
| Severe human suffering | 13 | 1.7 |
| Sudden violent death | 20 | 2.6 |
| Sudden accidental death | 85 | 11.0 |
| Serious injury, harm, or death you caused to someone else | 21 | 2.7 |
| Any other very stressful event or experience | 56 | 7.2 |
|  |  |  |
| Life events witnessed by the respondents |  |  |
| Natural disaster | 36 | 6.1 |
| Fire or explosion | 71 | 12.0 |
| Transportation accident | 55 | 9.3 |
| Serious accidents at work, home, or during | 62 | 10.4 |
| Exposure to toxic substance | 14 | 2.4 |
| Physical assault | 36 | 6.1 |
| Assault with a weapon | 36 | 6.1 |
| Sexual assault | 5 | 0.8 |
| Other unwanted or uncomfortable sexual experience | 6 | 1.0 |
| Combat or exposure to a warzone | 34 | 5.7 |
| Captivity | 5 | 0.8 |
| Life-threatening illness or injury | 42 | 7.1 |
| Severe human suffering | 70 | 11.8 |
| Sudden violent death | 68 | 11.4 |
| Sudden accidental death | 16 | 2.7 |
| Serious injury, harm, or death you caused to someone else | 38 | 6.4 |
| Any other very stressful event or experience | 36 | 6.1 |
| Traumatic events experienced |  |  |
| 0-10 | 180 | 79.6 |
| 11-21 | 46 | 20.4 |
|  |  |  |

**Employment status, income, and access to state benefits among veterans**: Details current and past employment, types of benefits received, ease of civilian job integration, and monthly income brackets.

**Supplementary Table 4** Employment and benefits

| Variables | Frequency | Percent |
| --- | --- | --- |
| **Employment*** | n=709 |  |
| Full time job | 347 | 48.9 |
| Part time job | 44 | 6.2 |
| Self employed | 13 | 1.8 |
| Government supported program | 1 | 0.1 |
| Unemployed and seeking | 7 | 1.0 |
| Unemployed and not seeking | 3 | 0.4 |
| Not working due to illness disability | 33 | 4.7 |
| Full time education | 5 | 0.7 |
| Part time education | 1 | 0.1 |
| Retired | 227 | 32.0 |
| Looking after the home | 3 | 0.4 |
| Other employment | 25 | 3.5 |
|  |  |  |
| **State benefits** |  |  |
| I don't know | 35 | 8.5 |
| No | 226 | 54.6 |
| Yes | 153 | 37.0 |
|  |  |  |
| **Types of state benefits*** | n=330 |  |
| Attendance allowance | 24 | 7.3 |
| Child benefit | 7 | 2.1 |
| Council Tax reduction | 30 | 9.1 |
| Disablement benefit | 15 | 4.5 |
| Housing benefit | 22 | 6.7 |
| Income support | 1 | 0.3 |
| Jobseeker’s allowance | 1 | 0.3 |
| Pension credit | 12 | 3.6 |
| Retirement allowance | 6 | 1.8 |
| State retirement pension | 122 | 37.0 |
| Statutory sick pay | 1 | 0.3 |
| Universal credit | 25 | 7.6 |
| Winter fuel payment | 49 | 14.8 |
| Working and child tax credits | 3 | 0.9 |
| Disability living allowance | 9 | 2.7 |
| I do not receive state benefits | 3 | 0.9 |
|  |  |  |
| **Monthly income** |  |  |
| Less than £1,000 | 46 | 11.1 |
| Between £1,000 and £1,500 | 89 | 21.5 |
| Between £1,500 and £1,900 | 75 | 18.1 |
| Between £1,900 and £2,300 | 63 | 15.2 |
| Between £2,300 and £2,750 | 55 | 13.3 |
| Between £2,750 and £3,160 | 28 | 6.8 |
| Between £3,160 and £3,580 | 20 | 4.8 |
| Between £3,580 and £4,000 | 13 | 3.1 |
| Between £4,000 and £4,250 | 9 | 2.2 |
| Over £4,250 | 16 | 3.9 |
|  |  |  |
| **Ease of finding employment after service** |  |  |
| Very easy | 148 | 35.7 |
| Fairly easy | 119 | 28.7 |
| Neither easy nor difficult | 44 | 10.6 |
| Fairly difficult | 44 | 10.6 |
| Very difficult | 40 | 9.7 |
| Not applicable | 16 | 3.9 |
| Don't know | 3 | 0.7 |
|  |  |  |
| **Time to find employment** |  |  |
| Less than three months | 282 | 68.1 |
| Three to five months | 50 | 12.1 |
| Six to eleven months | 37 | 8.9 |
| One to two years | 19 | 4.6 |
| Three to five years | 2 | .5 |
| More than five years | 1 | .2 |
| Not yet found work | 1 | .2 |
| Did not look for employment after service | 20 | 4.8 |
| Don't know | 2 | 0.5 |
|  |  |  |
| **Difficulty adapting to civilian job training** |  |  |
| Not at all difficult | 147 | 35.5 |
| Slightly difficult | 83 | 20.0 |
| Moderately difficult | 99 | 23.9 |
| Very difficult | 58 | 14.0 |
| Extremely difficult | 27 | 6.5 |
|  |  |  |
| **Military qualification recognized by employers** |  |  |
| Yes, always | 80 | 19.3 |
| Yes, usually | 91 | 21.9 |
| Yes, occasionally | 124 | 29.9 |
| No, never | 119 | 28.7 |
|  |  |  |
| **Use of any past military skills in present job** |  |  |
| None | 50 | 12.1 |
| A little | 75 | 18.1 |
| Quite a lot | 114 | 27.5 |
| Almost all | 64 | 15.5 |
| Don’t know | 0 | 0.0 |
| Not applicable | 111 | 26.8 |

*****Multiple responses required.

**Debt types and amounts reported by veterans**: Presents the frequency, sources, and scale of financial debts, including credit, personal loans, arrears, and written-off amounts.

**Supplementary Table 5** Debts

| Variables | Frequency | Percent |
| --- | --- | --- |
| **Debts^†^** |  |  |
|  | n=930 |  |
| Credit cards | 156 | 16.8 |
| Overdraft | 311 | 33.4 |
| Personal loans | 41 | 4.4 |
| Charge cards | 24 | 2.6 |
| Personal debts to family and friends | 20 | 2.2 |
| Doorstep collected loans | 14 | 1.5 |
| Credit sale agreements | 4 | 0.4 |
| Trading cheques and vouchers | 28 | 3.0 |
| Payday loan | 3 | 0.3 |
| Mortgage arrears repossession | 2 | 0.2 |
| Secured loan arrears repossession | 3 | 0.3 |
| Rent or mortgage arrears | 17 | 1.8 |
| Poll or council tax arrears | 7 | 0.8 |
| Fine default | 20 | 2.2 |
| Gas or electricity arrears | 5 | 0.5 |
| Maintenance arrears | 1 | 0.1 |
| Income tax arrears | 1 | 0.1 |
| VAT arrears distraint | 16 | 1.7 |
| Hire purchase arrears | 3 | 0.3 |
| Telephone arrears | 11 | 1.2 |
| Water bill arrears | 9 | 1.0 |
| Other | 225 | 24.2 |
| None | 8 | 0.9 |
| I prefer not to say | 1 | 0.1 |
|  |  |  |
| Other debts | n=225 |  |
| IVA | 2 | 0.9 |
| Benefit over payment (in appeal) | 1 | 0.4 |
| None presently, but at times it was a struggle | 1 | 0.4 |
| Holiday time share purchase | 1 | 0.4 |
| Owed on a CCJ will be clear nov 2024 | 1 | 0.4 |
| Universal overpayment/ rent arrears | 1 | 0.4 |
| Income tax non imprisonment | 1 | 0.4 |
| Personal loan (no Arrears) | 1 | 0.4 |
| Not specified | 216 | 96.0 |
|  |  |  |
|  |  |  |
| **Debt quantification*** | n=184 |  |
| Less than £1,000 | 29 | 15.8 |
| £1,000-£5,000 | 58 | 31.5 |
| £6,000-£10,000 | 30 | 16.3 |
| £11,000-£15,000 | 18 | 9.8 |
| £16,000-£20,000 | 9 | 4.9 |
| £21,000-£25,000 | 3 | 1.6 |
| £26,000-£30,000 | 5 | 2.7 |
| £31,000-£35,000 | 3 | 1.6 |
| £36,000-£40,000 | 1 | 0.5 |
| £41,000-£45,000 | 1 | 0.5 |
| £46,000-£50,000 | 1 | 0.5 |
| More than £50,000 | 1 | 0.5 |
| More than £100,000 | 2 | 1.1 |
| I'd rather not say | 23 | 12.5 |
|  |  |  |
| **Debts written off**** | n=407 |  |
| No | 399 | 96.4 |
| Yes | 8 | 1.9 |

**^†^** Multiple responses required

**Healthcare service utilisation among veterans:** Summarizes use of inpatient, GP, non-hospital, and additional health services, with frequency of contact and perceived health status.

**Supplementary Table 6** Healthcare utilisation

| Healthcare services | N | Percent |
| --- | --- | --- |
| **Inpatient services*** | N=236 |  |
| General medical ward | 18 | 8 |
| Emergency / crisis centre | 11 | 5 |
| Long-stay ward | 0 | 0 |
| Special hospital (e.g. Rampton) | 0 | 0 |
| Secure/semi-secure unit | 0 | 0 |
| Specialist assessment and/or treatment facility | 5 | 2 |
| Acute psychiatric ward | 0 | 0 |
| Rehabilitation ward/facility | 2 | 1 |
| None | 191 | 81 |
| Other | 9 | 4 |
|  |  |  |
| Number of contacts with inpatient days in the last 3 months |  |  |
| 0 | 216 | 95 |
| 1-5 | 6 | 3 |
| 6-10 | 5 | 2 |
|  |  |  |
|  | **Mean (SD)** |  |
| Inpatient days | 1.1 (0.3) |  |
|  |  |  |
| **GP services*** | N=389 |  |
| None | 59 | 15 |
| GP consultation in person | 107 | 28 |
| GP consultation by telephone | 93 | 24 |
| Practice nurse at GP surgery | 72 | 19 |
| Clinic provided by GP practice | 52 | 13 |
| GP out of hours service | 6 | 2 |
|  |  |  |
| Number of contacts with GP services in the last 3 months |  |  |
| 0 | 64 | 28 |
| 1-5 | 127 | 56 |
| 6-10 | 26 | 12 |
| >10 | 9 | 4 |
|  | **Mean (SD)** |  |
| Contact with GP services | 2.87 (3.99) |  |
|  |  |  |
|  |  |  |
| **Nonhospital based care*** | N=260 |  |
| Alternative medicine | 8 | 3 |
| Charity sector support | 12 | 5 |
| Community Nurse | 28 | 11 |
| Counsellor | 7 | 3 |
| None | 132 | 51 |
| NHS Direct/111 | 23 | 9 |
| Psychologist | 5 | 2 |
| District Nurse | 4 | 2 |
| Physiotherapist | 18 | 7 |
| Occupational Therapist | 4 | 2 |
| Other therapist | 5 | 2 |
| Walk in health service/Minor injuries | 14 | 5 |
|  |  |  |
| Number of contacts with non-hospital-based services in the last 3 months |  |  |
| 0 | 154 | 68.4 |
| 1-5 | 55 | 24.4 |
| 6-10 | 9 | 4.0 |
| >10 | 7 | 3.1 |
|  |  |  |
|  | **Mean (SD)** |  |
| Contact with non-hospital-based services | 1.4 (0.7) |  |
|  |  |  |
|  |  |  |
| **Additional services*** | N=233 |  |
| None | 202 | 87 |
| Alcohol Misuse Service | 2 | 1 |
| Caseworker’s mental health | 5 | 2 |
| Community mental health centre | 3 | 1 |
| Home help/home care worker | 3 | 1 |
| Self-help group, other support group or luncheon club | 12 | 5 |
| Social Worker | 3 | 1 |
| Home help/home care worker | 3 | 1 |
|  |  |  |
|  |  |  |
| **Perception of health (1-10)** |  |  |
| Mean | 6.31 |  |
| SD | 2.17 |  |

*Multiple responses

**Supplementary Table 7.** Multiple linear regression of mental health factors and gambling harm severity

|  | *B* | *SE* | *β* | *T* | *P* |
| --- | --- | --- | --- | --- | --- |
| Anxiety | .07 | .09 | .10 | 0.76 | .447 |
| Depression | .04 | .09 | .06 | 0.47 | .641 |
| PTSD | .01 | .05 | .03 | 0.28 | .781 |
| Loneliness | .26 | .16 | .16 | 1.62 | .106 |
| Alcohol use | .08 | .05 | .11 | 1.77 | .078 |
| Model summary  R  R^2^  Adjusted R^2^  F (0,5)  *p* | .34  .12  .10  5.87  <.001 |  |  |  |  |

Note. *B = unstandardised; SE* = Standard Error; *β* = standardised; *T* = t value; *P* = p value. p < .05; p < .001. All variables were log-transformed prior to analysis.
